# Dynamic Contrast-Enhanced Magnetic Resonance Imaging Findings and the Relevance of Histopathological Findings in Intraocular Solitary Fibrous Tumors

**DOI:** 10.7759/cureus.77299

**Published:** 2025-01-11

**Authors:** Miyuki Koga, Reiko Ideguchi, Ryota Kono, Akira L Yoshikawa, Ryo Toya

**Affiliations:** 1 Radiology, Nagasaki University Hospital, Nagasaki, JPN; 2 Radioisotope Medicine, Atomic Bomb Disease Institute, Nagasaki University, Nagasaki, JPN; 3 Ophthalmology, Nagasaki University Hospital, Nagasaki, JPN; 4 Pathology, Kameda Medical Center, Kamogawa, JPN; 5 Pathology, Nagasaki University Hospital, Nagasaki, JPN; 6 Radiation Oncology, Nagasaki University Hospital, Nagasaki, JPN

**Keywords:** case report, dynamic contrast-enhanced mri, extra-scleral extension, intraocular solitary fibrous tumors, intraocular tumor, stat6, time-intensity curve

## Abstract

Solitary fibrous tumors (SFT) are rare, and their intraocular occurrence is extremely uncommon. Therefore, magnetic resonance imaging (MRI) reports of intraocular SFT are limited, making preoperative diagnosis difficult. We present a case of primary choroidal SFT and explore dynamic contrast-enhanced (DCE) MRI findings along with histopathological findings. This investigation is expected to improve our understanding of the MRI findings of intraocular SFT. An 83-year-old woman presented with decreased vision in her right eye for several months. Computed tomography (CT) and MRI revealed a choroidal tumor with extension into the medial rectus muscle with suspected aggressive growth. The patient underwent right eye enucleation and recovered well after surgery. Histopathologically, diffuse STAT6 expression led to the final diagnosis of SFT. MRI findings typically expected for SFT were observed. T2-weighted images revealed heterogeneous signals with hyperintense and hypointense areas. DCE MRI revealed two patterns of time-intensity curves (TICs): marked enhancement and washout in T2-hyperintense areas and persistent enhancement in T2-hypointense areas. The intensity of T2-weighted imaging, enhancement pattern, and type of TICs in this case may be specific to intraocular SFT. An integrated evaluation of these findings may help differentiate SFT from other intraocular tumors.

## Introduction

Solitary fibrous tumors (SFT) are rare mesenchymal neoplasms initially reported as primary spindle cell tumors of the pleura. Previously, SFTs were considered distinct from hemangiopericytomas (HPCs). However, the expression of the NAB2-STAT6 fusion gene led to the histological classification as the same entity [1,2]. The 2016 World Health Organization (WHO) Classification of Tumors of the Central Nervous System describes this entity as SFT/HPC [3]. In 2021, the WHO revised the classification of SFT to encompass both SFT and HPC. The term “HPC” has been retired, with the tumor now termed SFT only [4]. The NAB2-STAT6 fusion gene is particularly expressed in SFT and is a significant component for diagnosing SFT [1]. The nuclear expression of STAT6 is consistent with that of the NAB2-STAT6 fusion gene and can serve as a highly specific substitute marker [1,2].

Although SFTs can occur anywhere in the body, intraocular SFTs are extremely rare, with only seven cases reported thus far [2,5-10]. Thus, these tumors are often misdiagnosed as other intraocular tumors such as choroidal hemangiomas, metastatic tumors, malignant melanomas, and amelanotic malignant melanomas [2,10,11]. There are no clear guidelines for the treatment of intraocular SFTs; however, enucleation is considered the primary treatment modality due to recurrence and metastasis risks [2,10]. Therefore, an accurate differentiation of SFTs from other tumors is crucial before initiating treatment. Magnetic resonance imaging (MRI) is useful for differentiation and can express the tissue properties and perfusion of lesions with contrast administration.

We describe a case of an intraocular SFT arising from the choroid and its MRI findings compared with histopathological findings.

## Case presentation

**Figure 1:**
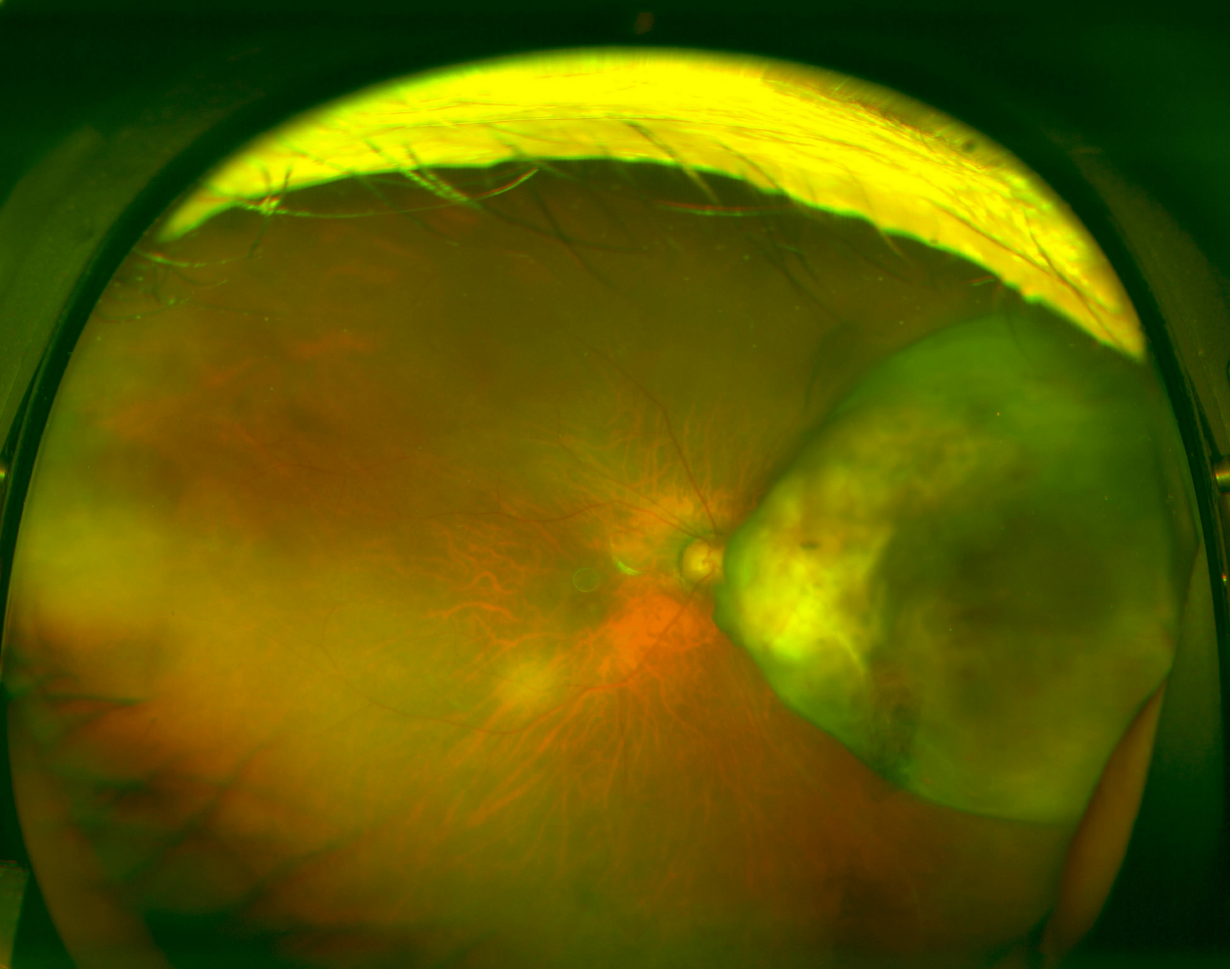
Photograph of the fundus This photograph shows a choroidal mass with elevation adjacent to the optic disc.

An 83-year-old woman was referred to our hospital for macular edema in the right eye. The patient complained of decreased vision in her right eye for several months. The patient had been medically treated for type 2 diabetes mellitus and hypertension but had no other history of ocular disease. Fundus examination revealed an elevated choroidal mass adjacent to the optic disc (Figure 1).

**Figure 2:**
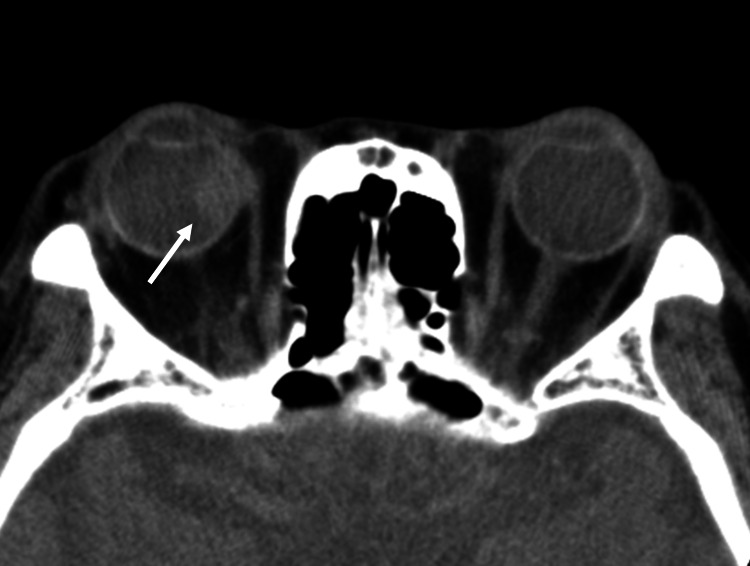
Axial CT scan CT reveals a well-circumscribed ovoid choroidal mass inside the right eyeball, measuring 8 × 12 × 11 mm (arrow). It was iso-attenuated compared to the lateral rectus muscle with infiltration into the internal rectus muscle, which was a suspected extra-scleral extension.

Computed tomography (CT) of the orbit revealed a lesion arising from the choroid into the vitreous, which appeared iso-attenuated compared with the lateral rectus muscle. The lesion was an oval and well-circumscribed choroidal mass, measuring 8 × 12 × 11 mm, with suspected extra-scleral extension (Figure 2).

**Figure 3:**
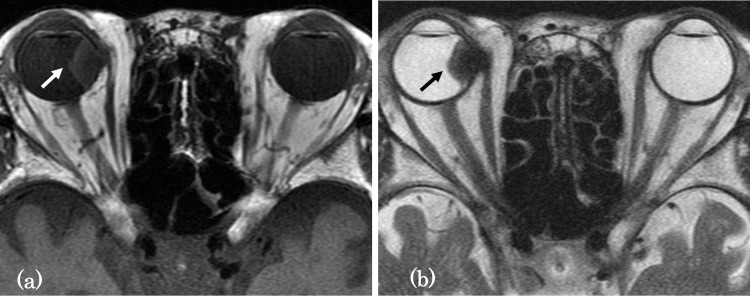
Axial MR images T1-weighted image (a) shows a choroidal mass with homogeneously isointense signal intensity compared with the cerebral cortex (white arrow). T2-weighted image (b) reveals that the mass is heterogeneous, mixed with hypointense and hyperintense signal intensities relative to the cerebral cortex (black arrow).

MRI revealed that the lesion had homogeneously isointense signal intensity compared with the cerebral cortex on T1-weighted images (Figure 3a). On T2-weighted images, the mass exhibited heterogeneous mixed hypointense and hyperintense signal intensities relative to the cerebral cortex (Figure 3b).

**Figure 4:**
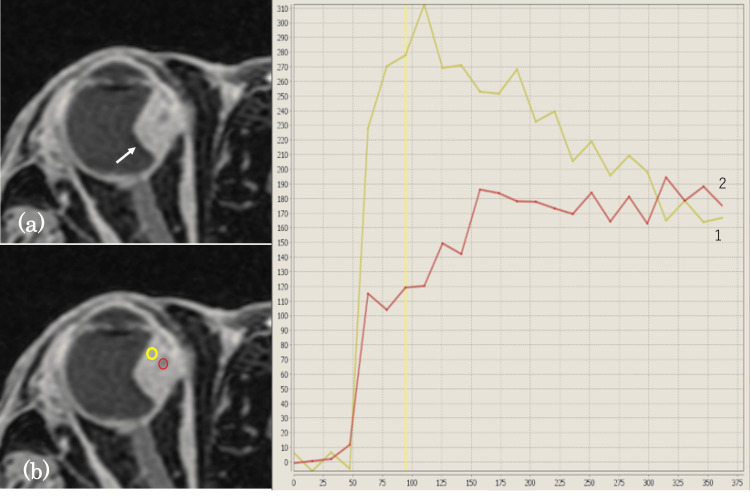
Axial DCE-MRI (a) and TICs (b) Axial DCE MRI (a) reveals that the mass has a heterogeneous enhancement (arrow). The TICs (b) are characterized by two types: early enhancement and washout (1) and persistent enhancement (2). 1 = margin of the mass (yellow ROI), 2 = center of the mass (red ROI). DCE-MRI: dynamic contrast-enhanced magnetic resonance imaging; TICs: time-intensity curves; ROI: region of interest

The lesion exhibited heterogeneous enhancement on contrast-enhanced MRI (Figure 4a). The hyperintense signal areas on T2-weighted images showed a marked enhancement and a washout pattern on TICs. The TIC of the hypointense signal areas on the T2-weighted images was characterized by a persistent enhancement curve (Figure 4b).

No evidence of metastasis was found by systemic evaluation. Considering that the patient was elderly and had underlying hypertension and diabetes mellitus, the patient was subsequently managed with observation alone. However, the mass increased in size, and the patient’s vision progressively worsened. Because of concerns regarding malignancy, the right eye was enucleated one year and five months after the patient’s initial examination.

**Figure 5:**
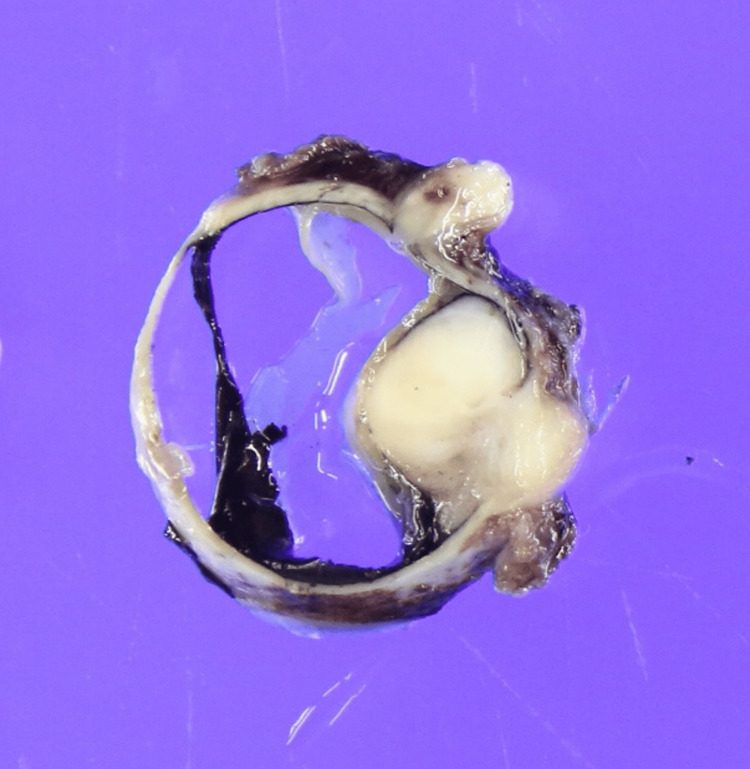
Cross-sectional view of the enucleated eye The image reveals a multinodular lesion, measuring 12.5 × 10 mm, expansively protruding from the sclera into the vitreous.

**Figure 6:**
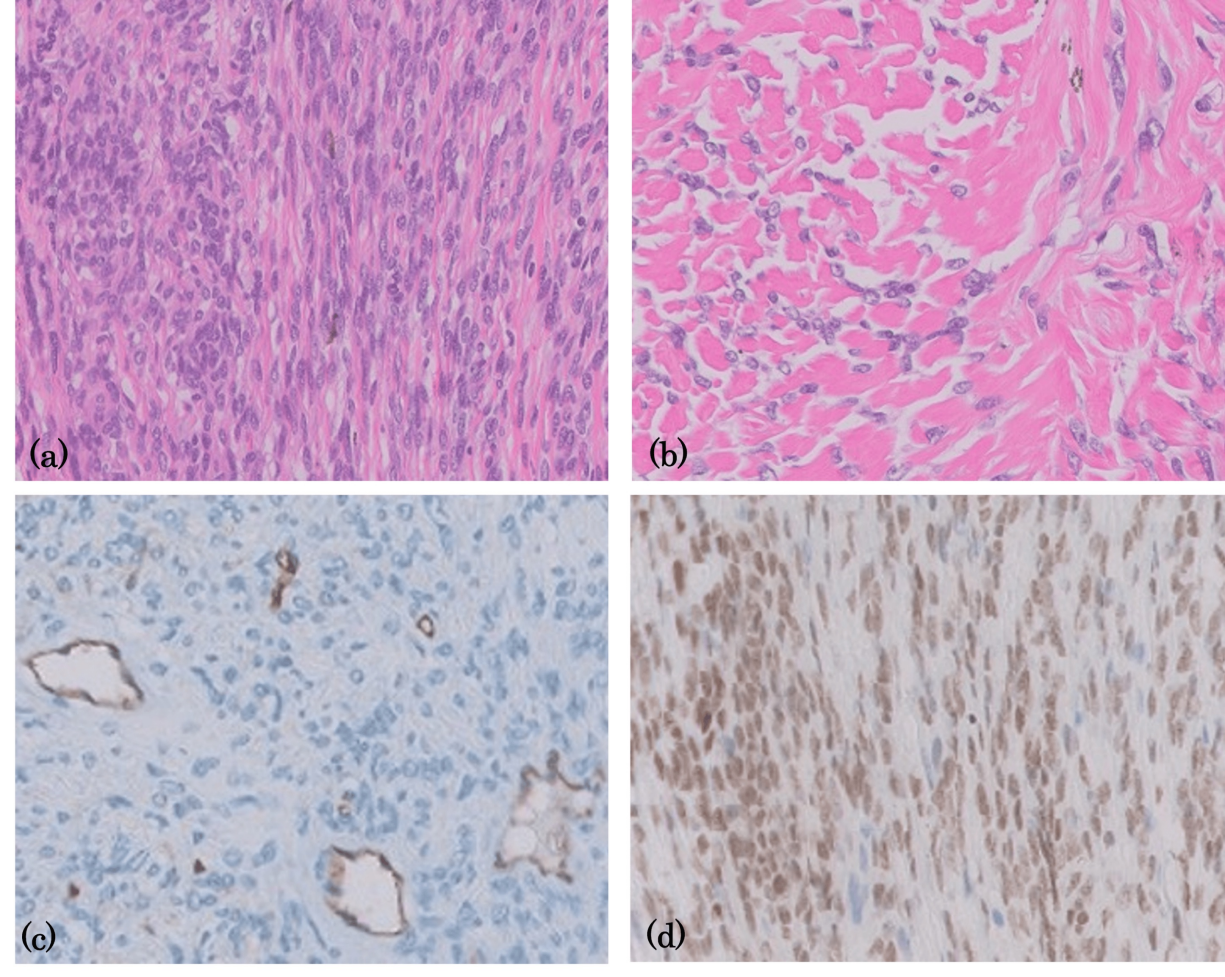
Hematoxylin and eosin (H&E) and immunochemical staining Spindle cell tumor with patternless growth of various densities, occasionally intervened by thick collagen fibers (a,b). Immunohistochemistry of CD34 highlights the sinusoidal vascular spaces lined by flattened endothelial cells (c). The tumor cells are diffusely positive for STA6 (d).

Figure 5 and Figure 6 show the histopathological findings. Gross sectioning of the right eye revealed a multinodular mass, measuring 12.5 × 10 mm. The mass protruded into the vitreous with extra-scleral extension (Figure 5).

Histological sections revealed dense fascicular proliferation of neoplastic spindle cells accompanied by a rich sinusoidal vasculature and a collagenous fiber background (Figure 6a and Figure 6b). The tumor appeared to expansively dissect the melanin-deposited layer of the choroid, suggesting that the tumor originated from the choroid. On immunohistochemical staining, the tumor cells were strongly and diffusely positive for CD34 (Figure 6c) and STAT6 (Figure 6d), consistent with SFT.

The patient was followed up regularly with CT and MRI postoperatively. No local recurrence or metastasis occurred after at least one year.

## Discussion

To the best of our knowledge, this is the eighth reported case of intraocular SFT and the second case with extra-scleral extension [2,5-10].

SFT occurs across various ages and at relatively equal frequencies in both sexes, with no observed racial differences. In seven cases, intraocular SFT occurred between the ages of 10 and 84 years and included three female and four male patients [2,5-10].

The symptoms of intraocular SFT vary widely and commonly include retinal detachment, intermittent eye pain, and unilateral visual dysfunction, with some cases being detected incidentally during intraocular examinations [2,5-10].

STAT6 immunohistochemical staining is a reliable method for confirming the diagnosis of SFT [1,2]. This is because it can prove the presence of the NAB-STAT6 fusion, which is specifically expressed in SFT [1,2].

Because reports on the imaging findings of intraocular SFT are limited, this case will describe those of general SFT alongside the relevant literature. According to the literature, SFT typically appears as an oval and well-defined soft tissue mass with isoattenuation relative to the extraocular muscle on CT [12]. MRI provides more detailed information. The previously reported MRI findings are summarized in Table 1.

**Table 1:** Summary of previously reported MRI findings of general SFT Sources:  [12-15] We compared and explored the imaging findings of the current intraocular case alongside the relevant literature; several features are consistent with the characteristics of general SFT. SFT: solitary fibrous tumors; TIC: time-intensity curve

Signal intensity or TIC patterns	
T1WI	Isointense
T2WI	Hypo-hyperintense, Flow-void
DCE-MRI	Early enhancement and washout pattern, Persistent pattern

On T1-weighted images, the signal intensity of the SFT is homogeneously isointense relative to that of the cerebral cortex [12,13]. On T2-weighted images, the lesion’s signal intensity varies, correlating with differences in the collagen fiber content and cellular components [12-14]. The literature indicates that the signal intensity on T2-weighted images decreases because of increasing collagen content [12,14]. Hyperintense signal areas are associated with internal hemorrhage, cystic degeneration, or relatively fresh fibrosis [12,13]. Flow-void signals are occasionally observed in areas of rapid blood flow [13].

MRI enhancement can sensitively indicate tumor tissue perfusion, and time-intensity curve (TIC) patterns can reveal detailed histological characteristics. Usually, the marked enhancement and washout pattern are specific to SFT, commonly occurring in very hyperintense areas on T2-weighted images [13-15]. This TIC pattern resembles that of the internal carotid artery, reflecting the high vascularity of SFTs, which is a notable feature of these tumors [12,13]. Furthermore, washout of contrast medium from tumors is correlated with the fibrous versus cellular stromata in tumors [15]. High-fibrous and low-cellularity stromata have a low washout ratio, whereas low-fibrous and high-cellularity stromata have a high washout ratio [15].

In this case, T2-weighted images and contrast-enhanced MRI revealed different signal intensities at the peripheral and central regions of the mass. Each of these imaging findings was consistent with those typically observed in general SFT. Furthermore, a flow void was not observed in this case.

The periphery of the mass exhibited a hyperintense signal on T2-weighted images and early enhancement and washout pattern on DCE-MRI. Pathologically, this area comprised numerous tumor cells and small sinusoid-like spaces. Thus, this signal pattern was considered to reflect the hypervascular, hypercellular, and low-fibrous organization of the tumor. On the other hand, the center of the mass exhibited hypointense signal intensity on T2-weighted images and persistent enhancement patterns on DCE MRI. Pathologically, the center of the mass contained abundant collagen fibers. This signal intensity can be attributed to the arrangement of high-fibrous and low-cellularity stromata [15].

The characteristic signal pattern observed in this case may be specific to intraocular SFT. Comparison of its findings with those of other intraocular tumors may help in the differential diagnosis.

## Conclusions

Intraocular SFTs are very rare and may be confused with other intraocular tumors. Individualized evaluations of the MRI findings for each tumor are necessary to distinguish these tumors. This report indicates that T2-weighted signal intensity and TIC patterns provide clues for precise diagnosis. These findings are clinically significant, and this report emphasizes the value of MRIs in making the correct diagnosis.
